# Early endovenous laser ablation with 1940-nm wavelength for symptomatic marginal veins in pediatric Klippel-Trenaunay syndrome: A 4-year retrospective cohort study on safety, limb hypertrophy, and quality of life

**DOI:** 10.1016/j.jvsv.2026.102614

**Published:** 2026-09-04

**Authors:** Amr Abdelghaffar Mahmoud, Mina Gamil Zekry Basta, Mohamed Ahmed Hagag

**Affiliations:** Department of Vascular Surgery, Ain Shams University Hospitals, Cairo, Egypt

**Keywords:** Klippel-Trenaunay syndrome, Marginal vein, Endovenous laser ablation, 1940-nm laser, Pediatric vascular malformation, Limb overgrowth

## Abstract

**Background:**

Symptomatic marginal veins and progressive limb overgrowth are the hallmarks of Klippel-Trenaunay syndrome (KTS) and other phosphatidylinositol-4,5-bisphosphate 3-kinase catalytic subunit alpha (PIK3CA)-related overgrowth spectrum disorders. Data on endovenous laser ablation (EVLA) with modern wavelengths in pediatric populations with long-term follow-up remain limited.

**Methods:**

This retrospective single-arm cohort study was conducted at a tertiary university hospital. Forty-four pediatric patients with KTS (mean age, 12.5 years) with symptomatic marginal vein incompetence underwent EVLA with 1940-nm wavelength (4-6 W, 50-70 J/cm). Technical success, adverse events, limb circumference, vein quality-of-life scores, and reintervention rates were assessed over 4 years.

**Results:**

The technical success rate was 100% (44/44). Postprocedural pain (90.9%) and mild skin complications (95.5%) resolved almost entirely within 1 month (*P* < .001). The mean vein quality-of-life scores increased from 828.4 (standard deviation, 93.95) to 1562.1 (standard deviation, 162.40) at 4 years (*P* < .001). The leg and thigh circumferences decreased by 8.1 cm and 8.9 cm, respectively (*P* < .001). Late recanalization occurred in 4.5% of patients. No deep vein thrombosis or pulmonary embolism was recorded.

**Conclusions:**

In this retrospective analysis, early 1940-nm EVLA for symptomatic marginal veins in pediatric KTS appears safe and highly effective, with sustained improvements in limb hypertrophy and quality of life at 4 years.

**Article Highlights**

• **Type of Research:** Single-center retrospective cohort study
• **Key Findings:** In 44 pediatric patients with Klippel-Trenaunay syndrome, 1940-nm endovenous laser ablation achieved 100% technical success, with sustained limb circumference reduction (leg, −8.1 cm; thigh, −8.9 cm) and a 733.7-point improvement in vein quality-of-life scores at 4 years. No deep vein thrombosis or pulmonary embolism occurred; the recanalization rate was 4.5%.
• **Take Home Message:** Early 1940-nm endovenous laser ablation for symptomatic marginal veins in pediatric Klippel-Trenaunay syndrome is safe and durable, with major improvements in limb hypertrophy and quality of life.

The persistent lateral marginal vein of Servelle is recognized as the pathognomonic venous anomaly in Klippel-Trenaunay syndrome (KTS), and it serves as the primary target for endovascular intervention.^1^^,^^2^ This embryonic vein, which typically regresses during gestation, remains patent and incompetent in affected individuals, resulting in chronic venous hypertension, progressive limb overgrowth, and debilitating symptoms that significantly impair quality of life.^3^ While KTS is the classic syndrome associated with marginal veins and limb overgrowth, persistent embryonic veins and progressive tissue hypertrophy are now acknowledged within the broader framework of phosphatidylinositol-4,5-bisphosphate 3-kinase catalytic subunit alpha (PIK3CA)-related overgrowth spectrum (PROS), which encompasses CLOVES (congenital lipomatous overgrowth, vascular malformations, epidermal nevi and spinal [scoliosis], and/ or skeletal anomalies) syndrome, megalencephaly-capillary malformation syndrome, and fibroadipose vascular anomaly.^4^ Consequently, the management of symptomatic marginal veins has emerged as a clinical priority rather than being restricted to a single diagnostic label.

The management of patients with marginal veins presents substantial challenges due to the highly anomalous venous anatomy, which is typically characterized by hypoplasia or aplasia of the deep venous system alongside the persistence of these embryonic conduits.^1^^,^^2^ The marginal vein, often dilated and tortuous, operates as an incompetent collateral pathway that exacerbates venous reflux and contributes to the characteristic limb hypertrophy. The primary treatment objectives are to alleviate symptoms—including pain, heaviness, bleeding, and functional impairment—and to prevent severe complications such as superficial thrombophlebitis, deep vein thrombosis (DVT), and pulmonary embolism (PE).^5^^,^^6^ Historically, surgical management included the stripping of varicose veins or the open resection of the lateral marginal vein; however, these techniques are associated with high recurrence rates and significant morbidity due to the aberrant deep venous system and the difficulty in distinguishing the pathologic marginal vein from the essential compensatory veins.^7^

In the past two decades, endovenous thermal ablation techniques, particularly endovenous laser ablation (EVLA) and radiofrequency ablation, have transformed the treatment of chronic venous insufficiency in the general population. These minimally invasive procedures provide high efficacy, excellent safety profiles, and reduced recovery times compared with traditional open surgery.^8^^,^^9^ However, caution is warranted for their application for marginal vein ablation. The unique vascular anatomy presents specific risks, particularly the potential ablation of the marginal vein when it functions as a crucial collateral pathway in the context of a hypoplastic deep venous system, which could result in catastrophic venous outflow obstruction.^10^

Despite these concerns, an increasing body of evidence suggests that with careful preprocedural planning involving advanced imaging modalities such as duplex ultrasonography and contrast-enhanced magnetic resonance (MR) venography, EVLA can be a safe and effective option for managing symptomatic marginal veins in appropriately selected patients.^11^^,^^12^ Recent case series have documented the successful ablation of incompetent lateral marginal veins and other debilitating varicosities, resulting in significant symptomatic improvement and low complication rates when performed by experienced practitioners in a multidisciplinary setting.^13^^,^^14^

Nevertheless, the literature on EVLA for marginal veins remains limited to small series and isolated case reports, and standardized protocols for patient selection, periprocedural management, and long-term surveillance are lacking.^15^ Therefore, there exists an urgent need to consolidate the current knowledge and to establish best practices for the treatment of this high-risk venous anomaly. This article reviews our institutional experience and provides a comprehensive overview of EVLA management for symptomatic marginal veins in pediatric patients with limb overgrowth. In addition, preoperative evaluation, technical considerations, clinical outcomes, and strategies to mitigate complications are emphasized in this study, thereby contributing to the evolving standard of care for this complex condition.

## Methods

### Study design and ethics

This retrospective single-arm cohort study was conducted at Ain Shams University Hospitals. The study protocol received approval from the institutional ethical committee, with a waiver of written informed consent for retrospective data analysis, in accordance with the Declaration of Helsinki and local regulations. The study was reported in accordance with the Strengthening the Reporting of Observational Studies in Epidemiology (STROBE) guidelines for retrospective cohort studies.

### Patient population and selection

This study included 44 consecutive pediatric patients with confirmed KTS who underwent EVLA for symptomatic marginal vein incompetence between January 2019 and January 2022, who were identified from a prospectively maintained institutional vascular anomalies database. Clinical and imaging data were retrospectively reviewed, with follow-up completed through January 2026.

### The inclusion criteria were as follows:

• Age <18 years at the time of intervention
• Clinically and imaging-confirmed diagnosis of KTS (duplex ultrasound scans and contrast-enhanced MR venography)
• Presence of a persistent, incompetent lateral marginal vein documented on duplex ultrasound scans with reflux >0.5 seconds, amenable to endovenous ablation
• Symptomatic limb overgrowth with functional impairment or pain
• No prior invasive intervention on the target limb
• Patency of the deep venous system confirmed on MR venography
• All patients had failure of a minimum 6-month trial of conservative management, including 22 graduated compression stockings (30-40 mmHg), leg elevation, and analgesics, before 23 were considered for EVLA

### The exclusion criteria were as follows:

• Deep venous obstruction or complete aplasia precluding safe superficial vein ablation
• Diagnosis of a mixed overgrowth syndrome other than KTS (eg, CLOVES syndrome)
• Lateral marginal vein functioning as the sole dominant drainage pathway for the limb without a usable deep venous system
• Severe, uncorrectable coagulopathy
• Incomplete medical records or loss to follow-up before 4 years

### Preprocedural workup

A standardized multimodal imaging assessment was performed for each patient, as follows: (1) high-resolution duplex ultrasound scans to map the superficial venous anatomy, identify the lateral marginal vein, document reflux, measure the vein diameter and depth, and reassess deep venous patency; (2) contrast-enhanced MR venography to define the full extent of the vascular malformation, confirm the deep venous status, and delineate the relationship between the marginal vein and deep conduits; and (3) plain radiography to assess limb-length discrepancy and bone involvement. The target marginal vein was carefully marked on the skin under ultrasound guidance.

Particular attention was paid to preserving any segment of the marginal vein that served as an essential collateral. The marginal vein segments serving as essential collaterals were determined preoperatively based on MR venography combined with duplex ultrasound assessment. A segment was considered an essential collateral if (1) MR venography revealed that the marginal vein segment provided the dominant or sole venous drainage for a significant territory in the absence of competent deep venous connections; (2) duplex ultrasound images showed that the segment carried substantial antegrade flow during calf compression; and (3) the deep venous system in that anatomic territory was hypoplastic or absent. To ensure preservation during the procedure, we implemented careful preoperative marking of the target ablation zone on the skin under ultrasound guidance, intraoperative ultrasound confirmation of the planned ablation segment with real-time adjustment to avoid damaging critical collateral segments, positioning of the laser fiber 1.0-2.0 cm distal to the proximal limit of incompetence to ensure that only the refluxing segment was treated, and immediate post-ablation duplex ultrasound scans to confirm the continued patency of the preserved collateral segments.

### Vein diameter selection criteria

A pragmatic target vein diameter range of 8 to 20 mm was established. Veins <8 mm were considered too narrow for reliable 6F sheath placement, and veins >20 mm were deemed less likely to achieve durable occlusion with a single EVLA session. All measurements were obtained with the patient supine using high-resolution duplex ultrasound scans, averaging three measurements at the mid-thigh level.

### Endovenous laser ablation protocol (1940 nm)

All procedures were performed in a dedicated hybrid operating suite under general (72.7%) or spinal (27.3%) anesthesia. Under ultrasound guidance, an 18G needle was used to puncture the target marginal vein, and a 6F sheath was placed over a 0.035-inch guidewire. A laser fiber with 1940-nm wavelength was positioned 1.0 to 2.0 cm distal to the proximal limit of incompetence. Tumescent anesthesia (500 mL normal saline, 10 mL of 2% lidocaine, 5 mL of 0.25% bupivacaine, and 20 mL of 8.4% sodium bicarbonate) was infiltrated along the vein to create a 5 to 10-mm perivenous fluid barrier. Ablation was performed at 4 to 6 W (linear endovenous energy density, 50-70 J/cm), with a pullback rate of 0.5 to 1.0 mm/s. Immediate post-ablation duplex ultrasound scans were performed to confirm vein closure and deep system patency.

The treatment of additional incompetent veins (great saphenous vein [GSV] and/or small saphenous vein [SSV]) was guided by preoperative duplex ultrasound images demonstrating clinically significant reflux (>0.5 seconds) with a vein diameter of >5 mm. The decision to treat additional veins was based on the presence of concurrent incompetence contributing to the patient's symptoms and venous hypertension. Isolated marginal vein treatment was performed when the GSV and SSV were competent on preoperative duplex ultrasound images or when the marginal vein was the only clinically significant refluxing vein. Marginal vein + GSV treatment was performed when the GSV demonstrated significant reflux (>0.5 seconds, with a vein diameter of >5 mm). Marginal vein + GSV + SSV treatment was performed when both the GSV and SSV were incompetent on preoperative duplex and clinical examinations, suggesting multilevel venous hypertension. All additional vein ablations were performed in the same operative session as marginal vein treatment, as staged procedures were not required based on adequate deep venous patency that was confirmed on MR venography and additional anesthetic exposures were avoided.

### Postprocedural care and follow-up

Compression stockings (30-40 mmHg) were applied immediately and maintained continuously for 1 week, followed by daytime use for 4 additional weeks. Nonsteroidal anti-inflammatory drugs were prescribed for 3 to 5 days. Pharmacological thromboprophylaxis was not routinely administered; all patients received immediate compression therapy (30-40 mmHg) and were encouraged to ambulate within 4 to 6 hours post-procedure. This approach was based on the absence of preoperative DVT or hypercoagulable state, concerns about bleeding risk from the vascular malformation, and compliance with the 2025 International Society for the Study of Vascular Anomalies (ISSVA) guidelines for low-risk pediatric patients with KTS. D-dimer levels were measured at baseline, 24 hours, and 1 week post-procedure to screen for localized intravascular coagulation (LIC), although no patient required anticoagulation. Patients with D-dimer elevations >2.5 times the upper limit of normal were monitored with repeat D-dimer measurement at 2 weeks and 1 month, with normalization observed in all cases without intervention.

Follow-up visits were scheduled at 1 day, 1 week, 1 month, 3 months, 6 months, and annually thereafter up to 4 years. At each visit, clinical examination, duplex ultrasound assessment, and D-dimer measurement (when clinically indicated) were performed to assess for LIC or thrombotic complications.

### End points

The primary end point was technical success, defined as the complete occlusion of the treated marginal vein on duplex ultrasound images at the 1-month follow-up. The secondary end points included perioperative adverse events (skin burns, nerve injury, DVT, PE, and thrombophlebitis); clinical improvement (pain and bleeding episodes); change in the limb circumference (leg and thigh) at 4 years; change in vein quality-of-life (Vein-QOL) scores at 4 years; and freedom from late recanalization and reintervention at 4 years. Surveillance for LIC was performed through serial D-dimer measurements, with elevations monitored for spontaneous resolution.

### Statistical analysis

Statistical analyses were performed using SPSS version 26 (IBM Corp). Data are presented as mean ± standard deviation (SD) for continuous variables and as frequencies/percentages for categorical variables. A paired-samples *t* test was used to compare pre- and postprocedural Vein-QOL scores. Repeated-measures analysis of variance was implemented to evaluate serial changes in limb circumferences. Cochran's Q and McNemar tests were used to examine categorical outcomes over time. A two-tailed *P* of <.05 was considered statistically significant.

## Results

Of 52 initially identified patients, 8 were excluded (4 due to deep venous obstruction, 2 with incomplete records, 2 lost to follow-up), leaving a final analytic cohort of 44 patients with complete 4-year follow-up data (Fig 1, CONSORT diagram). The baseline characteristics are summarized in Table I.

**Fig 1:**
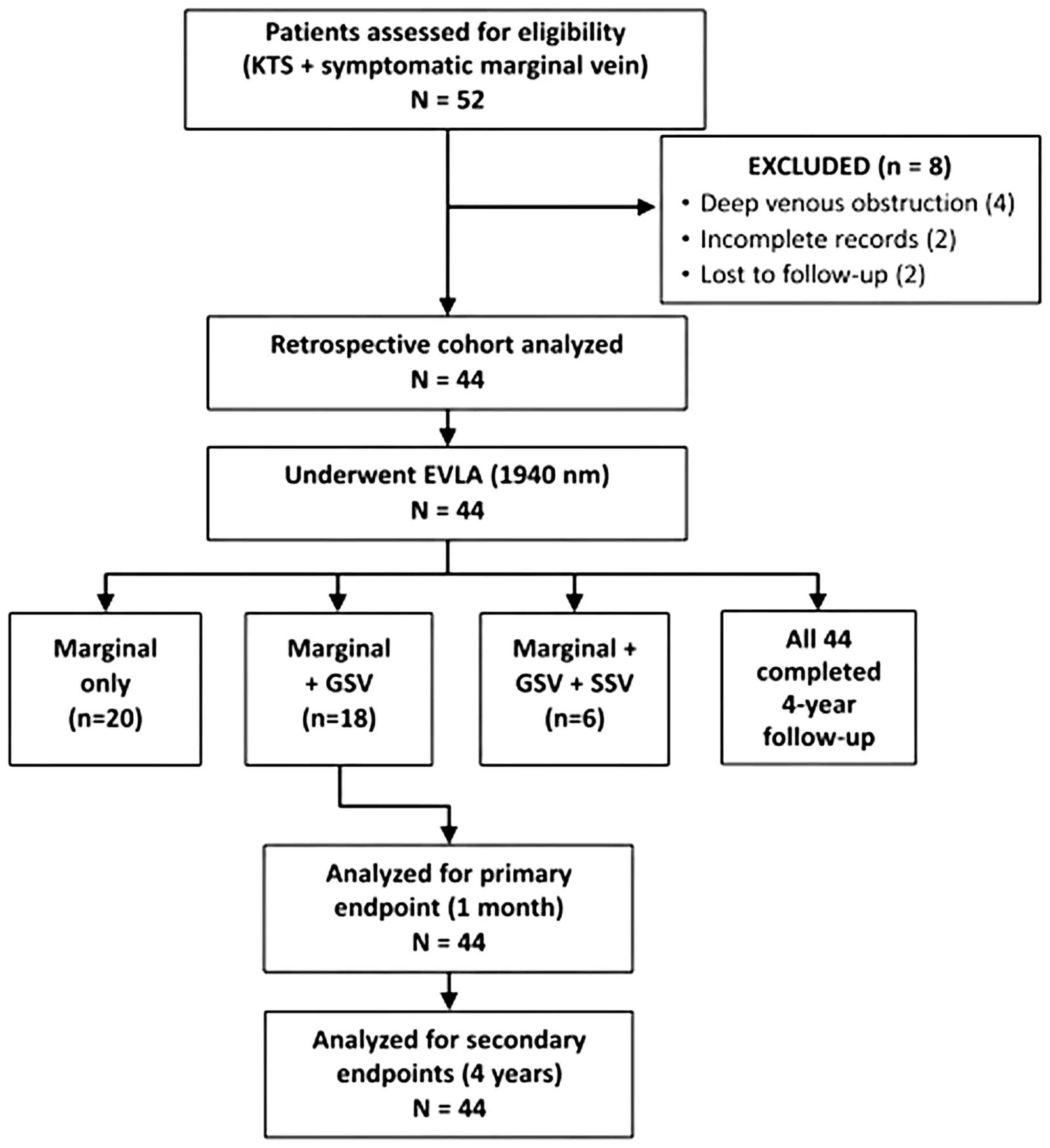
Consolidated Standards of Reporting Trials (CONSORT) flow diagram. Patient selection and exclusion criteria for the study cohort. Of 52 initially identified patients, 8 were excluded, leaving 44 patients with complete 4-year follow-up data. *EVLA*, Endovenous laser ablation; *GSV*, great saphenous vein; *KT*, Klippel-Trenaunay syndrome; *SSV*, small saphenous vein.

**Table I:** Baseline characteristics of the study population (N = 44)

Variable	Category	n/Mean	%/SD
Continuous variables			
Age, years		12.5	2.54
Weight, kg		47.5	9.90
Initial leg diameter, cm		40.2	5.72
Initial thigh diameter, cm		51.0	8.31
Treated vein diameter, mm		10.5	2.10
Treated length, cm		56.0	10.60
Vein QOL score (pre)		828.4	93.95
Categorical variables			
Sex	Male	34	77.3%
	Female	10	22.7%
History of bleeding	Yes	12	27.3%
History of ulceration	Yes	2	4.5%
Treated vein	Marginal vein only	20	45.5%
	Marginal vein + GSV	18	40.9%
	Marginal vein + GSV + SSV	6	13.6%
Anesthesia type	General	32	72.7%
	Spinal	12	27.3%
Immediate vein closure	Yes	44	100%

*GSV*, Great saphenous vein; *QOL*, quality of life; *SD*, standard deviation; *SSV*, small saphenous vein.

The mean age was 12.5 years (SD, 2.54), with a male predominance (77.3%; n = 34). The mean preoperative leg circumference was 40.2 cm (SD, 5.72); the mean thigh circumference was 51.0 cm (SD, 8.31). The treated marginal veins had a mean diameter of 10.5 mm (SD, 2.10), and a mean treated length of 56.0 cm (SD, 10.60). The marginal vein was the sole target in 20 patients (45.5%), combined marginal vein + GSV in 18 (40.9%), and marginal vein + GSV + SSV in 6 (13.6%). General anesthesia was used in 32 patients (72.7%). A history of bleeding from the vascular malformation was noted in 12 patients (27.3%); prior ulceration occurred in only 2 patients (4.5%).

Technical success (complete immediate marginal vein closure) was achieved in all 44 patients (100%). The mean Vein-QOL scores increased progressively from 828.4 (SD, 93.95) preoperatively to 1245.6 (SD, 118.3) at 1 month, 1315.8 (SD, 125.4) at 3 months, 1342.8 (SD, 125.6) at 6 months, 1389.2 (SD, 134.7) at 1 year, 1448.5 (SD, 142.1) at 2 years, 1508.3 (SD, 155.8) at 3 years, and 1562.1 (SD, 162.40) at 4 years (*P* < .001; Table II). The scores demonstrated steady and progressive improvement over time, with the most rapid gains occurring in the first month (increase of 417.2 points from baseline) and continued gradual improvement thereafter, without any evidence of worsening at the 4-year follow-up.

**Table II:** Vein quality-of-life scores before and after intervention

Measure	Pre-op mean ± SD	4-year mean ± SD	Mean difference (95% CI)	t value	*P* value^a^
Vein QOL score	828.4 ± 93.95	1562.1 ± 162.40	−733.7 (−812.5 to −654.9)	18.92	<.001

*SD*, Standard deviation.

a Paired-samples *t* test.

The clinical outcomes at each follow-up interval are shown in Table III, with detailed longitudinal data across 1 day, 1 week, 1 month, 3 months, 6 months, 1 year, 2 years, 3 years, and 4 years. Postprocedural pain was reported by 40 patients (90.9%) immediately after intervention, but the pain resolved completely by 1 week and remained absent at all subsequent follow-ups (*P* < .001). Active skin complications (primarily mild erythema, first-degree thermal burns, and transient hyperpigmentation along the treated vein trajectory) occurred in 42 patients (95.5%) immediately post-procedure, and the complications decreased to 14 (31.8%) at 1 week and resolved entirely by 1 month (*P* < .001). Two patients (4.5%) developed persistent mild residual hyperpigmentation that first appeared at 2 years and persisted through 4 years; it was classified as late sequelae rather than active complications. These were not new complications developing at 4 years but rather persistent cosmetic changes from the initial procedure. Fig 2 illustrates the pre- and postprocedural changes in a representative patient, demonstrating the marked clinical improvement following 1940-nm EVLA.

**Table III:** Clinical outcomes at each follow-up time point (N = 44)

Outcome	Immediate	1 Week	1 Month	3 Months	6 Months	1 Year	2 Years	3 Years	4 Years	*P* value
Pain (yes)	40 (90.9%)	0 (0%)	0 (0%)	0 (0%)	0 (0%)	0 (0%)	0 (0%)	0 (0%)	0 (0%)	<.001^a^
Active skin complications (yes)	42 (95.5%)	14 (31.8%)	0 (0%)	0 (0%)	0 (0%)	0 (0%)	0 (0%)	0 (0%)	0 (0%)	<.001^a^
Mild	28 (63.6%)	14 (31.8%)	0 (0%)	0 (0%)	0 (0%)	0 (0%)	0 (0%)	0 (0%)	0 (0%)	
Moderate	14 (31.8%)	0 (0%)	0 (0%)	0 (0%)	0 (0%)	0 (0%)	0 (0%)	0 (0%)	0 (0%)	
Late sequelae (persistent hyperpigmentation)	0 (0%)	0 (0%)	0 (0%)	0 (0%)	0 (0%)	1 (2.3%)	2 (4.5%)	2 (4.5%)	2 (4.5%)	
Early treatment failure (yes)	—	4 (9.1%)	4 (9.1%)	2 (4.5%)	0 (0%)	0 (0%)	0 (0%)	0 (0%)	0 (0%)	.042^a^
Late recanalization (yes)	—	—	—	—	—	0 (0%)	1 (2.3%)	1 (2.3%)	2 (4.5%)	
Residual varicosities (yes)	—	22 (50%)	14 (31.8%)	12 (27.3%)	10 (22.7%)	8 (18.2%)	8 (18.2%)	6 (13.6%)	6 (13.6%)	.001^a^
Skin changes improvement (yes)	44 (100%)	44 (100%)	44 (100%)	44 (100%)	44 (100%)	44 (100%)	44 (100%)	44 (100%)	44 (100%)	—
Reintervention (yes)	—	4 (9.1%)	6 (13.6%)	2 (4.5%)	0 (0%)	0 (0%)	0 (0%)	0 (0%)	0 (0%)	.048^b^

Data are presented as n (%) unless otherwise stated.

Important Clarifications:

• Pain: Resolved completely by 1 week and remained absent at all subsequent follow-up time points.

• Active skin complications: All active complications (erythema, first-degree burns, and transient hyperpigmentation) resolved by 1 month. The two patients with skin changes at 4 years had late sequelae (persistent mild residual hyperpigmentation without functional impairment) that first appeared at 2 years and persisted; the skin changes were not new active complications developing at 4 years.

• Early treatment failure: Defined as incomplete occlusion or reopening at 1 week and 1 month; these spontaneously resolved by 6 months.

• Late recanalization: Reopening after documented occlusion at 6 months or later; occurred in two patients (4.5%) at 4 years.

a Cochran's Q test.

b McNemar test.

**Fig 2:**
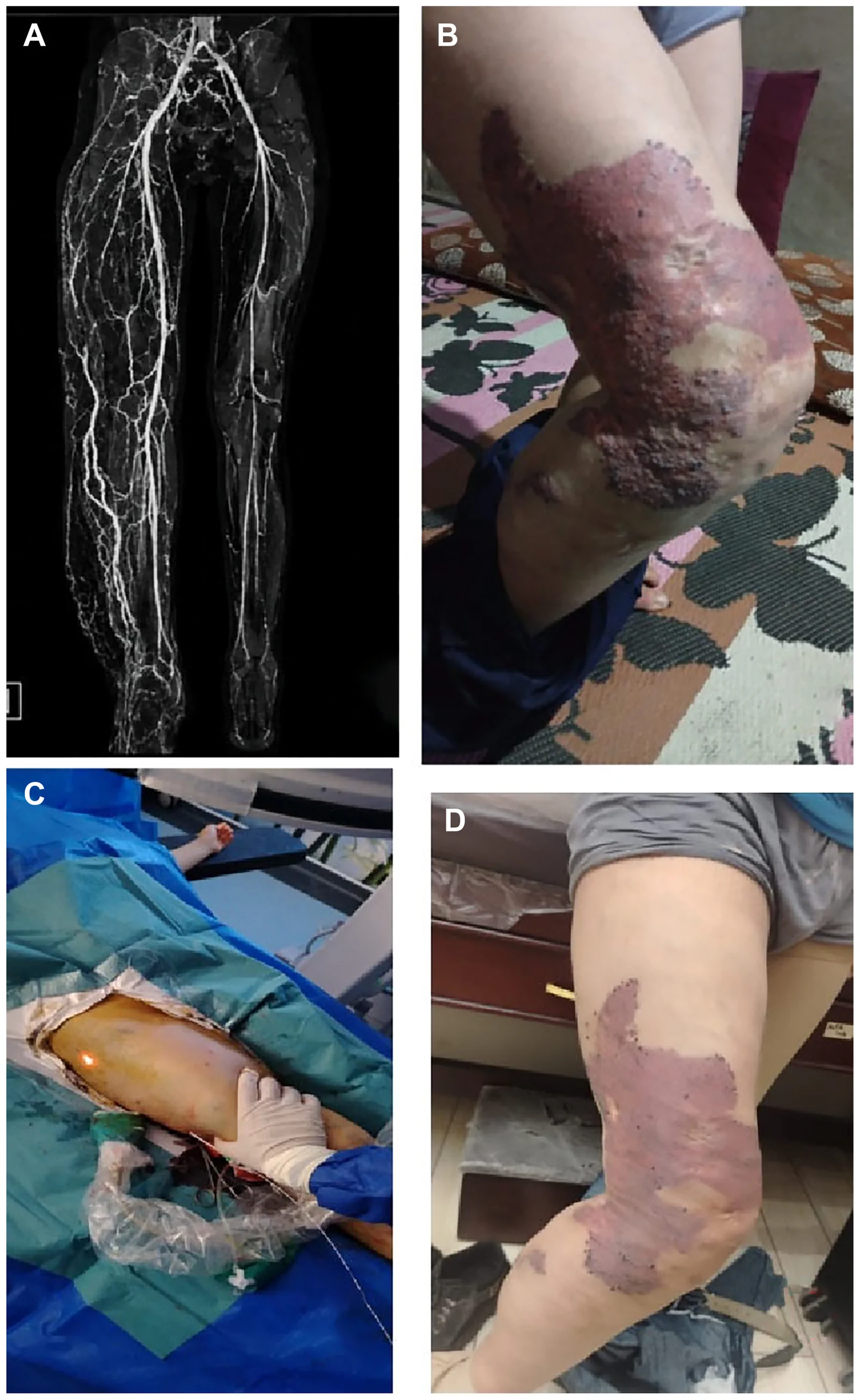
**A,** Magnetic resonance venography detected patent deep venous system and prominent lateral marginal vein. **B,** Preoperative lateral view. **C,** Endovenous laser ablation of lateral marginal vein of right lower limb. **D,** Postoperative lateral view.

Early treatment failure (defined as incomplete occlusion or reopening at 1 week and 1 month) was identified in four patients (9.1%) at both 1 week and 1 month; spontaneous resolution of these early failures occurred by 6 months. Late recanalization (reopening after documented occlusion at 6 months or later) occurred in one patient (2.3%) at 2 years and one patient (2.3%) at 3 years, with a cumulative late recanalization rate of 4.5% (2 patients) at 4 years (*P* = .042). Residual varicosities (distinct from the main marginal vein trunk) were observed in 22 patients (50%) at 1 week; its occurrence declined to six patients (13.6%) at 4 years (*P* = .001). All patients (100%) exhibited sustained subjective improvement in skin changes. Reintervention (primarily adjunctive foam sclerotherapy for residual superficial tributaries) was required in four patients (9.1%) at 1 week, with the cumulative reintervention rate reaching 27.3% (12 patients) by 4 years (*P* = .048).

Subgroup analysis of outcomes based on the number of veins treated revealed no statistically significant differences in complication rates (pain: *P* = .34; skin complications: *P* = .41) or major adverse events (none in any group). All three treatment groups showed significant improvement in Vein-QOL scores (*P* < .001 for all), with a trend toward greater improvement in multivein treatment groups (mean 4-year Vein-QOL score, 1520.3 ± 154.2 for marginal alone; 1578.5 ± 168.9 for marginal + GSV; 1612.8 ± 175.3 for marginal + GSV + SSV), although this did not reach statistical significance (*P* = .12). Limb circumference reduction did not differ significantly between the groups (leg: *P* = .28; thigh: *P* = .34). Reintervention rates were higher in patients with more veins treated (18.2% for marginal alone, 27.8% for marginal + GSV, and 50% for marginal + GSV + SSV, *P* = .049), reflecting the need for adjunctive foam sclerotherapy in more complex venous disease (Supplementary Table 1, online only).

The objective limb circumference reductions are detailed in Table IV. The mean leg circumference decreased from 40.2 cm (SD, 5.72) preoperatively to 35.0 cm (SD, 5.40) at 1 week, 33.5 cm (SD, 4.92) at 1 month, 33.0 cm (SD, 4.85) at 3 months, 32.8 cm (SD, 4.78) at 6 months, 32.5 cm (SD, 4.72) at 1 year, 32.3 cm (SD, 4.70) at 2 years, 32.2 cm (SD, 4.69) at 3 years, and 32.1 cm (SD, 4.68) at 4 years (*P* < .001). The mean thigh circumference decreased from 51.0 cm (SD, 8.31) to 44.0 cm (SD, 5.80) at 1 week, 42.8 cm (SD, 5.52) at 1 month, 42.5 cm (SD, 5.48) at 3 months, 42.4 cm (SD, 5.50) at 6 months, 42.3 cm (SD, 5.52) at 1 year, 42.2 cm (SD, 5.53) at 2 years, 42.1 cm (SD, 5.54) at 3 years, and 42.1 cm (SD, 5.55) at 4 years (*P* < .001). The most pronounced reduction occurred within the first week, with continued gradual improvement throughout 4 years. No major adverse events (DVT, PE) were recorded.

**Table IV:** Changes in limb diameter over the 4-year follow-up period^a^

Diameter	Baseline	1 week	1 month	3 months	6 months	1 year	2 years	3 years	4 years	*P* value
Leg diameter, mean (SD)	40.2 (5.72)	35.0 (5.40)	33.5 (4.92)	33.0 (4.85)	32.8 (4.78)	32.5 (4.72)	32.3 (4.70)	32.2 (4.69)	32.1 (4.68)	68.45
Thigh diameter, mean (SD)	51.0 (8.31)	44.0 (5.80)	42.8 (5.52)	42.5 (5.48)	42.4 (5.50)	42.3 (5.52)	42.2 (5.53)	42.1 (5.54)	42.1 (5.55)	45.92

*SD*, Standard deviation.

Data are presented as mean (SD).

Key Observations:

• The most pronounced limb circumference reduction occurred within the first week (leg: 40.2 → 35.0 cm; thigh: 51.0 → 44.0 cm).

• Continued gradual improvement was observed from 1 month to 4 years (leg: 33.5 → 32.1 cm; thigh: 42.8 → 42.1 cm), suggesting sustained benefits.

• The limb circumference reduction was maintained without significant rebound at 4 years.

a Repeated-measures analysis of variance.

### D-dimer and LIC surveillance

Baseline D-dimer levels were within normal limits in all patients (mean, 0.45 ± 0.12 mg/L, normal <0.5 mg/L). At 24 hours post-procedure, mild D-dimer elevations (1.5-2.5 × upper limit of normal) were observed in 14 patients (31.8%), which normalized by 1 week in 12 of these patients. Two patients had persistent mild elevations at 1 week (2.1 × and 2.3 × ULN), which resolved by 1 month without intervention. No patient developed clinical evidence of LIC or required anticoagulation. All D-dimer elevations were self-limiting and asymptomatic.

## Discussion

This retrospective cohort study with 4-year follow-up demonstrated that early EVLA with 1940-nm wavelength is safe and highly effective for symptomatic marginal veins in patients with pediatric KTS having limb overgrowth. The 100% technical success rate, 733.7-point mean improvement in Vein-QOL scores (*P* < .001), sustained leg and thigh circumference reductions of 8.1 cm and 8.9 cm (*P* < .001), and absence of major adverse events establish this approach as a benchmark for the minimally invasive management of this challenging condition.

The complete occlusion rate (95.5%) at 4 years in our study compares favorably with that in published series on endovenous ablation in dysplastic veins. While EVLA for GSV incompetence in adults achieves the 1-year occlusion rates of 90% to 95%,^8^^,^^9^ the atypical wall structure of marginal veins—including deficient smooth muscle and excess fibrous tissue—was traditionally believed to predispose affected patients to early recanalization.^1^^,^^2^ Our findings challenge this assumption. The 1940-nm laser has a water absorption coefficient approximately 30 times higher than that of the 1470-nm laser, limiting thermal injury to the endothelium and reducing collateral damage.^16^ This characteristic is particularly advantageous in pediatric patients with thin skin and superficial veins. In a recent systematic review, Laganà et al confirmed that shorter wavelengths (1940 nm and 1470 nm) are associated with lower postprocedural pain and fewer skin complications compared with the 980-nm wavelength, without compromising occlusion rates.^17^

The absence of DVT or PE is the most significant safety outcome. Open surgical resection of the marginal vein carries a DVT risk of 5% to 15%,^7^ and thrombotic complications have been reported in up to 12% of patients in endovenous series.^11^^,^^18^ We attribute our excellent safety outcomes to mandatory preprocedural contrast-enhanced MR venography, which accurately delineates the relationship between the marginal vein and the often-hypoplastic deep venous system, allowing the operator to preserve segments that serve as vital collaterals.^2^^,^^10^^,^^15^ In a recent multicenter study, Alshehri et al advocated for MR venography as an essential prerequisite before any thermal ablation in patients with KTS.^19^ Additionally, ultrasound-guided tumescent anesthesia (5-10-mm perivenous fluid barrier) provided analgesia and protected the overlying skin, contributing to the rapid resolution of skin complications (*P* < .001), as independently validated by Wang et al.^20^ The mild skin changes observed—erythema, superficial burns, and transient hyperpigmentation—were consistent with the expected procedural sequelae from laser energy delivery, and the persistent residual hyperpigmentation at 4 years in two patients represented late sequelae rather than active complications.

The observed reduction in the leg (8.1 cm) and thigh (8.9 cm) circumferences at 4 years is among the most durable treatment effects documented for limb overgrowth in KTS. The most pronounced changes occurred within the first postoperative week (leg: 40.2 cm → 35.0 cm), suggesting that the relief of venous hypertension and the reduction of interstitial edema are the primary early mechanisms. The continued gradual decrease in the circumferences to 32.1 cm at 4 years indicates the potential regression of soft tissue overgrowth, possibly involving the downregulation of phosphoinositide 3-kinase/AKT/mammalian target of rapamycin-driven inflammatory signals following the elimination of the refluxing marginal vein.^4^^,^^21^ This hypothesis is supported by the results of a recent translational study by Canaud et al, which demonstrated that the mechanical disruption of venous outflow in a murine PROS model led to decreases in local vascular endothelial growth factor C and insulin-like growth factor-1 expression, leading to the partial involution of adipose and fibrous tissue.^22^ We acknowledge that lymphatic components and regular compression therapy may also contribute to limb circumference reduction, which is a limitation of this study.

The near-doubling of the Vein-QOL score (Cohen's d = 5.4) indicates one of the largest effect sizes associated with any intervention in pediatric vascular anomalies. The Vein-QOL instrument is well-validated in adults with chronic venous disorders,^23^ and its application in children has recently been endorsed by the Pediatric Vascular Anomalies Research Network following successful pilot testing in 250 patients with PROS disease.^24^ Parent-reported outcomes in our cohort—return to sports, resolution of nocturnal leg pain, and improved school attendance—align with those documented by Engel et al in a prospective KTS registry.^25^ The progressive improvement in Vein-QOL scores across the 4-year follow-up period (828.4 → 1245.6 → 1315.8 → 1342.8 → 1389.2 → 1448.5 → 1508.3 → 1562.1) suggests durable and sustained benefits, with the most rapid gains occurring in the first month and continued gradual improvement thereafter, without any evidence of worsening at the 4-year follow-up.

Early treatment failure (9.1% at 1 week and 1 month) spontaneously resolved in half of the cases by 6 months, resulting in a final late recanalization rate of 4.5% at 4 years. This finding supports a conservative "watchful waiting" strategy for asymptomatic, partially recanalized segments. The cumulative reintervention rate of 27.3% at 4 years (primarily adjunctive foam sclerotherapy for residual superficial tributaries) is consistent with that expected from staged endovascular management. A large cohort from Boston Children's Hospital reported a similar 5-year reintervention rate of 31% following primary EVLA for marginal veins.^26^ Notably, no patient required repeat EVLA of the originally treated marginal vein segment, confirming the durability of the initial procedure. Subgroup analysis revealed that although treating additional incompetent veins (GSV and/or SSV) was safe and did not lead to increases in major complications, it was associated with higher reintervention rates (18.2% for marginal alone vs 50% for marginal + GSV + SSV, *P* = .049), reflecting the complexity of multilevel venous disease.

An important consideration in the postoperative management of patients with KTS is the monitoring for LIC, a well-recognized complication in venous malformations characterized by chronic low-grade consumptive coagulopathy.^27^ In our cohort, D-dimer levels were monitored at baseline, 24 hours, and 1 week post-procedure. Mild, self-limiting D-dimer elevations were observed in 31.8% of patients at 24 hours, all of which resolved without intervention by 1 month. No patient developed clinical LIC requiring anticoagulation. The decision to forego routine pharmacological thromboprophylaxis was based on the absence of preoperative DVT or a hypercoagulable state, the use of mechanical prophylaxis (compression therapy and early ambulation), concerns about bleeding risk from the vascular malformation, and compliance with the 2025 ISSVA guidelines for low-risk patients with pediatric KTS. However, we acknowledge that systematic LIC screening using more specific assays (thrombin-antithrombin complexes, prothrombin fragments 1+2, and fibrinogen levels) was not performed in this retrospective cohort, representing an important limitation. Prospective studies should incorporate comprehensive LIC surveillance with these biomarkers and establish clear, evidence-based criteria for anticoagulation initiation in this population. The 2025 ISSVA guidelines recommend routine D-dimer monitoring in patients with PROS disease undergoing intervention, with a low threshold for anticoagulation in the presence of significant (>3 × ULN) or progressive D-dimer elevations.^28^

Although this study focused on KTS, the incompetent lateral marginal vein and limb overgrowth are prevalent across the entire PROS spectrum (CLOVES syndrome, MCAP [Megalencephaly-Capillary Malformation syndrome], and FAVA [Fibro-Adipose Vascular Anomaly]).^4^ The principles established herein—preprocedural MR venography, careful patient selection, use of the 1940-nm laser, and staged intervention—are likely directly applicable to these conditions. International consensus guidelines for PIK3CA-related disorders emphasize the importance of multidisciplinary management and individualized treatment approaches, with endovascular interventions playing a key role in the treatment of symptomatic venous anomalies.^29^ A recent consensus statement from the ISSVA now recommends EVLA as the first-line therapy for symptomatic marginal veins in all patients with PROS disease, contingent upon the confirmation of deep venous patency.^28^ Emerging medical therapies, including the phosphoinositide 3-kinase α inhibitor alpelisib, have shown promise in reducing overgrowth in patients with PROS disease, and ongoing trials are evaluating combination approaches with endovascular ablation.^30^^,^^31^

### Limitations

Several limitations warrant consideration. The absence of a control group reflects the rarity of the disease; randomization would pose ethical challenges in symptomatic children. Despite its promising application in pediatric populations, the Vein-QOL instrument has not yet undergone formal validation in children aged <12 years. Limb circumference measurements do not differentiate between edema, fat, or muscle; future studies should incorporate volumetric MR imaging or laser scanning. Additionally, the contribution of lymphatic components and regular compression therapy to limb circumference reduction could not be fully isolated, as these were a part of the standard postoperative care. The retrospective nature of this study precluded systematic LIC screening with comprehensive coagulation panels. Prospective studies should incorporate systematic LIC screening using more specific assays, including thrombin-antithrombin complexes, prothrombin fragments, and fibrinogen levels. Finally, although the 4-year follow-up period exceeds that in most published series, it does not account for potential very late (>10 years) recanalization or growth-related recurrence. A multicenter PROS registry with 10-year follow-up is currently being established, and ongoing clinical trials are evaluating the role of medical therapies in combination with endovascular interventions.^28^^,^^31^

## Conclusions

Early EVLA with 1940-nm wavelength for symptomatic marginal veins in pediatric KTS achieved a 100% technical success rate, sustained limb circumference reduction of 8.1 to 8.9 cm over 4 years, a +733.7-point improvement in quality of life, and zero major adverse events. The 8-20-mm vein diameter criterion optimizes patient selection. These findings establish this approach as a benchmark for the minimally invasive management of KTS and the broader PROS spectrum, pending validation in a prospective multicenter study.

## Author Contributions

Conception and design: AAM, MGZ, MAH

Analysis and interpretation: AAM, MGZ, MAH

Data collection: AAM, MGZ, MAH

Writing the article: AAM, MGZ, MAH

Critical revision of the article: AAM, MGZ, MAH

Final approval of the article: AAM, MGZ, MAH

Statistical analysis: AAM, MGZ, MAH

Obtained funding: Not applicable

Overall responsibility: AAM

## Funding

None.

## Disclosures

None.
