# Supplementary material for: Early endovenous laser ablation with 1940-nm wavelength for symptomatic marginal veins in pediatric Klippel-Trenaunay syndrome: A 4-year retrospective cohort study on safety, limb hypertrophy, and quality of life
Source: J Vasc Surg Venous Lymphat Disord. 2026 Sep 4;15(1):102614. doi: 10.1016/j.jvsv.2026.102614 (PMC13694763; doi:10.1016/j.jvsv.2026.102614)
Supplement: Supplementary file 1 [file mmc1.docx]

**Table S1. Subgroup Analysis by Number of Veins Treated**

| **Outcome** | **Marginal Vein Only (n=20)** | **Marginal + GSV (n=18)** | **Marginal + GSV + SSV (n=6)** | **p value** |
| --- | --- | --- | --- | --- |
| **Pain at 1 week** | 0 (0%) | 0 (0%) | 0 (0%) | 1.00 |
| **Skin complications at 1 week** | 6 (30.0%) | 6 (33.3%) | 2 (33.3%) | 0.41 |
| **Early treatment failure at 1 month** | 2 (10.0%) | 1 (5.6%) | 1 (16.7%) | 0.56 |
| **Late recanalization at 4 years** | 1 (5.0%) | 0 (0%) | 1 (16.7%) | 0.38 |
| **Vein-QOL score at 4 years, mean (SD)** | 1520.3 ± 154.2 | 1578.5 ± 168.9 | 1612.8 ± 175.3 | 0.12 |
| **Leg circumference reduction at 4 years (cm)** | 8.0 ± 1.2 | 8.2 ± 1.4 | 8.1 ± 1.1 | 0.28 |
| **Thigh circumference reduction at 4 years (cm)** | 8.7 ± 1.5 | 9.0 ± 1.6 | 8.9 ± 1.4 | 0.34 |
| **Cumulative reintervention at 4 years** | 4 (18.2%) | 5 (27.8%) | 3 (50.0%) | 0.049 |
| **Major adverse events (DVT/PE)** | 0 (0%) | 0 (0%) | 0 (0%) | 1.00 |

Data are presented as n (%) unless otherwise stated.
GSV = great saphenous vein; SSV = small saphenous vein; DVT = deep vein thrombosis; PE = pulmonary embolism.

**Key Findings:**

- No significant differences in complication rates between groups.
- All three groups showed significant Vein-QOL improvement (p < 0.001 for all).
- Trend toward greater Vein-QOL improvement in multi-vein treatment groups (not statistically significant, p = 0.12).
- Higher reintervention rates in patients with more veins treated (p = 0.049), reflecting greater complexity of multilevel venous disease.
